# Post-exercise consumption of milk or Greek yogurt may support the anti-inflammatory environment following high-intensity exercise

**DOI:** 10.3389/fspor.2026.1911130

**Published:** 2026-08-26

**Authors:** Emily C. Fraschetti, Nicholas Cheng, Monika Nguyen, Christopher G. R. Perry, Ali A. Abdul-Sater, Andrea R. Josse

**Affiliations:** School of Kinesiology and Health Science, Faculty of Health, Muscle Health Research Centre, York University, Toronto, ON, Canada

**Keywords:** cytokines, dairy products, exercise, inflammation, milk, nutrition, yogurt

## Abstract

**Introduction:**

Following exercise, there is a transient increase in circulating inflammatory mediators (e.g., cytokines). This acute inflammatory response may be modulated by post-exercise nutrition. Dairy products contain many anti-inflammatory constituents that may favourably modulate exercise-induced inflammation. This study aimed to investigate how different post-exercise nutrition strategies [milk, Greek yogurt [GY], carbohydrate [CHO], or water [no nutrition]] modulate the exercise-induced inflammatory response.

**Methods:**

Twenty healthy recreationally active adults (12/8 F/M; age=23 ± 4.0 years; BMI: 22 ± 2.3 kg/m^2^) were recruited to participate in this four-arm crossover. Participants completed a high-intensity, multi-modal exercise bout consisting of plyometrics, resistance exercise and high-intensity cycling sprints. Post-exercise supplements (milk, GY, CHO, or water) were provided 5 m and 1 h post-exercise and before bed. Standardized meals and snacks were provided to participants on the day of exercise. Blood samples were collected pre-exercise, 5 m, 1 h, 4 h, and 24 h post-exercise, and plasma circulating cytokine concentrations were evaluated.

**Results:**

Following exercise, IL-1ra and IL-8 increased while IFN-*γ* decreased, regardless of post-exercise nutrition (time effects). IL-6 increased post-exercise, returning to baseline at 4 h and 24 h for males and females, respectively (time*sex interaction). TNF was higher in MILK vs. all other trials at 24 h (time*trial interaction). IL-10 was lower in CHO at 24 h vs. WATER and MILK (time*trial interaction). Further, MILK and GY had higher IL-10 at 4 h vs. CHO and WATER. The ratio of TNF/IL-10 was lower in WATER at 1 h (vs. MILK and CHO), and lower in MILK and GY at 4 h (vs. CHO) (time*trial interaction).

**Discussion:**

Together, these results illustrate that dairy consumption increases IL-10 at 4 h post-exercise (greater than CHO and WATER), leading to a more anti-inflammatory TNF/IL-10 ratio (vs. CHO), which may relate to improved exercise recovery time.

## 1 Introduction

A single bout of acute exercise, particularly high-intensity exercise (1–4), induces a transient inflammatory response (5, 6). This inflammatory response has been well characterized in the literature and is often simplified into two phases, an initial pro-inflammatory phase followed by a secondary anti-inflammatory phase (5, 6). Exercise-induced muscle damage is the main trigger for this inflammatory response, as it primarily serves to clear damaged tissue and repair the muscle (5, 6). During the pro-inflammatory phase, immune cells are mobilized to the area of damage, and there is increased production of pro-inflammatory cytokines such as interleukin (IL)-6, IL-8, tumor necrosis factor (TNF), and IL-1β, ultimately aiding in clearing damaged tissue (5, 6). This process also produces reactive oxygen species and proteases from immune cells that can often result in secondary damage to the muscle (5–7). Secondary damage refers to additional muscle damage that occurs following the damaging exercise, resulting from immune cell infiltration and the release of cytotoxic compounds from immune cells (6, 8, 9). As such, the pro-inflammatory phase can worsen muscle damage and delay the onset of the second, anti-inflammatory phase (i.e., the muscle repair and regeneration phase) (6, 8). The second phase, or anti-inflammatory phase, is characterized by an increase in anti-inflammatory cytokines, particularly IL-10 (5, 6), which aid in promoting muscle repair and regeneration. While these inflammatory responses are taking place at the site of muscle damage, inflammatory cytokines can spill over into the circulation (10, 11). Thus, examining systemic markers of inflammation may provide insight into the local inflammatory response over time.

Balancing these two phases of exercise-induced inflammation is crucial in optimizing recovery and adaptation to exercise (12–14). Importantly, the inflammatory response following exercise is a crucial part of the muscle’s adaptation to exercise, so the goal is not to abolish this response, as this could result in delayed recovery or attenuated muscle adaptation (12, 15). However, modulating this response (i.e., decreasing the pro-inflammatory phase while increasing the anti-inflammatory phase) may support adaptation while improving recovery time (13–17). Indeed, there are many strategies used to attenuate and/or modulate the inflammatory response to exercise and improve recovery, including cryotherapy, massage, stretching, heat, non-steroidal anti-inflammatory drugs, and nutrition (13, 15–17). In terms of nutrition, dairy products, because of the nutrients they contain, such as protein and vitamin D, are of particular interest in supporting exercise-induced adaptations and promoting recovery (13, 18–21).

Dairy products (i.e., milk, yogurt and cheese) are complex, nutrient-dense wholefoods containing varying amounts of high-quality protein (whey and casein, to support muscle protein synthesis), carbohydrates (primarily lactose, to support glycogen resynthesis), electrolytes (to support rehydration), and shortfall vitamins and minerals (e.g., vitamin D and calcium, to support bone health) (13, 18–24). Dairy products also contain anti-inflammatory constituents (25), which may influence exercise-induced inflammation. Indeed, components of whey protein (e.g., lactoferrin) and micronutrients, such as vitamin D, selenium and zinc, present in dairy products (vitamin D fortified in milk), have been shown to have anti-inflammatory properties (25). Importantly, not all dairy products are equal (26, 27). Within a wholefood, nutrients may act synergistically, and the action/impact of nutrients within a wholefood matrix may be greater than, or different from, the actions of the nutrients in isolation (28). Milk and Greek yogurt (GY) are both dairy products, but they differ in their physical and nutritional matrices and processing methods. Milk has a liquid food form, while GY has a semi-solid food form, resulting in faster gastric emptying following milk consumption than GY (26). GY is a fermented food, while milk is not. Fermented dairy products, like GY, have been shown to elicit greater anti-inflammatory effects vs. non-fermented dairy products (29). GY contains less whey and more casein than milk due to whey being strained during processing (30, 31). In Canada and other countries worldwide, milk must be fortified with vitamin D (32, 33), which has known immunomodulatory properties (25). As such, different dairy products may elicit differential effects on exercise-induced inflammation.

Few studies have been primarily designed to assess the influence of dairy consumption on the exercise-induced inflammatory response (34–36). However, in the absence of measuring inflammation, several studies have shown that post-exercise milk consumption improves muscle soreness (18, 37–40), subsequent performance (39, 41–47) and strength metrics (18, 37–39, 43, 44). Nonetheless, some studies have investigated the influence of dairy protein (48–61), plain (i.e., white) milk (36, 38, 40, 43, 44) and flavoured (i.e., chocolate or strawberry milk) (34, 35) on exercise-induced inflammation. While many studies show no added benefit of post-exercise consumption of dairy protein (whey and/or casein) on exercise-induced inflammation (48–50, 54, 56, 58–61), two studies have observed an enhanced IL-10 response following post-exercise whey protein consumption compared to a water (52) or a carbohydrate (CHO) control (53). While some studies have investigated the influence of post-exercise milk consumption, plain or flavoured, on exercise-induced inflammation (34–36, 40, 43, 44), many of these studies only examined one inflammatory mediator (40, 43, 44). Investigation of multiple markers of inflammation is key to understanding the overall inflammatory response, as the immune system is highly interconnected and these cytokines can directly up- and down-regulate one another (62). Studies investigating flavoured milk have not observed differences vs. CHO and water controls (34, 35). However, it may still be prudent to investigate the use of plain milk, as the higher glycemic response of chocolate milk may be masking anti-inflammatory effects (63). Our lab has previously illustrated divergent IL-10 responses following post-exercise CHO and milk consumption (36). Further, relative changes in cytokines 48 h post-exercise revealed lower concentrations of IL-1*β*, IL-6 (trending), and IL-10 following MILK vs. CHO (36). Taken together, whey protein or milk consumption post-exercise may modulate the IL-10 response following exercise. As IL-10 is an important trigger for the onset of the anti-inflammatory phase of exercise-induced inflammation, investigation of wholefood dairy products, like milk and GY, during these mid-hours of recovery is warranted. Furthermore, given the popularity of yogurt, over milk, in Canada (64) and the fermented nature of yogurt (29), investigation of how GY influences exercise-induced inflammation is prudent.

Thus, the objective of this study was to investigate the influence of four differential acute post-exercise supplements: milk, GY, CHO, and water (i.e., no nutrition control) on the systemic cytokine response to high-intensity exercise in healthy, young adults. Specifically, we examined the circulating concentrations of IL-1β, IL-1 receptor antagonist (IL-1ra), IL-6, IL-8, IL-10, TNF, and interferon-*γ* (IFN-*γ*). We hypothesized that the dairy arms (milk and GY) would improve exercise-induced inflammation, resulting in a more anti-inflammatory environment, compared to CHO and water. Further, while both dairy arms would elicit an anti-inflammatory response, we hypothesized that the fermented GY would be more anti-inflammatory than milk.

## 2 Materials and methods

Twenty young, healthy, university-aged males (*n* = 8) and females (*n* = 12) were recruited for the “Bone and Inflammatory Outcomes—Nutrition and Exercise” (BIONEX) trials. This study was approved by the York University Research Ethics Board (ORE #2019–045) and was registered at https://www.clinicaltrials.gov (NCT06041087). Recruitment for this study took place in Toronto (ON, Canada), and the study ran from October 2023 to February 2025.

### 2.1 Participants

Participants were recruited using approved posters, word of mouth, and social media posts. Interested participants underwent a virtual screening visit, which included answering basic health questions with a research coordinator to ensure their eligibility for the study. Inclusion criteria included: 1) aged 18–31 years, 2) normal BMI (18.5–24.9 kg/m^2^), and 3) no to low exercise level (i.e., recreationally active, ≤2 structured exercise sessions/week). Participants were excluded if they, 1) had any medical conditions that may affect primary outcomes (specifically, inflammatory, autoimmune, and/or musculoskeletal conditions), 2) were current smokers (including cigarettes, marijuana, and vaping), 3) were consuming vitamin D and/or calcium supplements, and 4) had diagnosed allergies to dairy protein and/or lactose intolerance. Participants who were deemed eligible were invited to the lab for a familiarization session.

### 2.2 Study design

This study utilized a randomized, crossover experimental design, whereby participants underwent four trial conditions consisting of a high-intensity exercise bout and differential post-exercise nutrition. The four trial arms were: milk (MILK), Greek yogurt (GY), carbohydrate (CHO), and water (WATER), which served as a no-nutrition control. Prior to the start of the study, conditions were coded 1–4 by an individual not involved in the study and input into a random number generator to determine randomization for 25 participants. Trials were separated by a minimum 2-week washout for male participants and females on hormonal birth control (*n* = 3). To control for menstrual cycle, naturally cycling females (*n* = 9) had a minimum 4-week washout, and were tested in the early follicular phase of their cycle. Prior to each trial, participants were asked to avoid taking non-steroidal anti-inflammatory drugs, and refrain from alcohol consumption and participation in exercise for 12 h, 24 h, and 48 h before the trial, respectively.

### 2.3 Familiarization session

At the familiarization session, participants were provided a comprehensive walkthrough of the study by a researcher. Upon obtaining written, informed consent, participants completed the 2018 Physical Activity Readiness Questionnaire (PAR-Q+) to ensure they were safe to perform exercise and the Godin-Shepard Leisure-Time Physical Activity Questionnaire to confirm their weekly activity level. It was confirmed that all participants had low habitual exercise levels with most participants reporting they never/rarely perform high or moderate intensity exercise. While some participants reported they sometimes or often performed high or moderate intensity exercise, this mainly comprised of brisk walking. Body composition was assessed and estimated 1-repetition maximum (e1RM) testing and an incremental cycling test were conducted.

#### 2.3.1 Body composition

Body fat percentage was determined using air displacement plethysmography via the Bod Pod (COSMED USA Inc., Concord, CA, USA). Before each participant arrived to the lab, the device and integrated scale were calibrated. To ensure accurate readings, participants were instructed to wear tight-fitting clothing, remove shoes and all jewelry, and were provided with a swim cap to compress their hair. While in light clothing and without shoes, weight was measured using the calibrated, integrated scale for the Bod Pod system. Two measurements were taken, and if they differed by 150 mL, a third measurement was obtained. Measurements were averaged and used to calculate body density using the Siri equation (65). Height was measured using a stadiometer and reported to the nearest 0.1 cm.

#### 2.3.2 Estimated 1 repetition maximum (e1RM) testing

Following a dynamic warm-up, a certified personal trainer (N. Cheng) conducted e1RM testing for the leg press, chest press, and seated row using selectorized resistance exercise machines. For each exercise, participants were shown proper form and technique to mitigate the risk of injury. All e1RM tests began with three warm-up sets consisting of 8 repetitions with increasing loads aimed to have participants working at a rating of perceived exertion of 3 (easy), 5 (somewhat difficult), and 7 (hard), respectively, using a modified Borg Category-Ratio (CR10) Scale of Perceived Exertion (66). Following the warm-up sets, participants performed their “working sets” with a maximum of 5 repetitions. These working sets were designed to bring participants closer to their e1RM and ensure volitional failure within 3–4 sets and ≤5 repetitions. Rest between sets during e1RM testing was determined by participant readiness. Once volitional failure was achieved within 5 repetitions, their 1RM was estimated using the Brzycki equation (67). Verbal encouragement was provided to all participants during e1RM testing.

#### 2.3.3 Incremental cycling test

To determine peak power output (PPO), participants took part in an incremental cycling test on a stationary cycle ergometer (Monark 894E, Monark Sports & Medical, Vansbro, Sweden). Participants were fitted with a chest strap heart rate monitor (Polar H10, Polar Electro Oy, Kempele, Finland) to evaluate heart rate. Each test began with a 4-minute warm-up at 50–70 revolutions per minute (RPM), with only the weight of the empty cycle basket [1 kg equivalent to 50–70 watts (W)]. This cadence/RPM was maintained throughout the test. Their RPM was individualized based on their body weight in kg, rounded down to the nearest 10 kg (i.e., a participant weighing 68 kg pedaled at 60 RPM). Following the warm-up, the resistance was increased by 0.5 kg every minute, which corresponded to 25–35 W depending on their cycling RPM (W = load * RPM). Resistance was increased each minute until the participant could no longer maintain the correct RPM for a full minute. Verbal encouragement was provided to all participants during cycling testing. The Watts achieved in the last fully completed minute were deemed to be their PPO. Heart rate was recorded during the final 10 s of the warm-up and each minute of the incremental test to verify participants were working at maximal work rates [>90% estimated heart rate max; %HRMax; as determined by 220 beats/minute—age in years (68)]. A cool-down of active recovery (relaxed cycling with no resistance or walking) followed the incremental test.

### 2.4 Experimental trial

**Figure 1:**
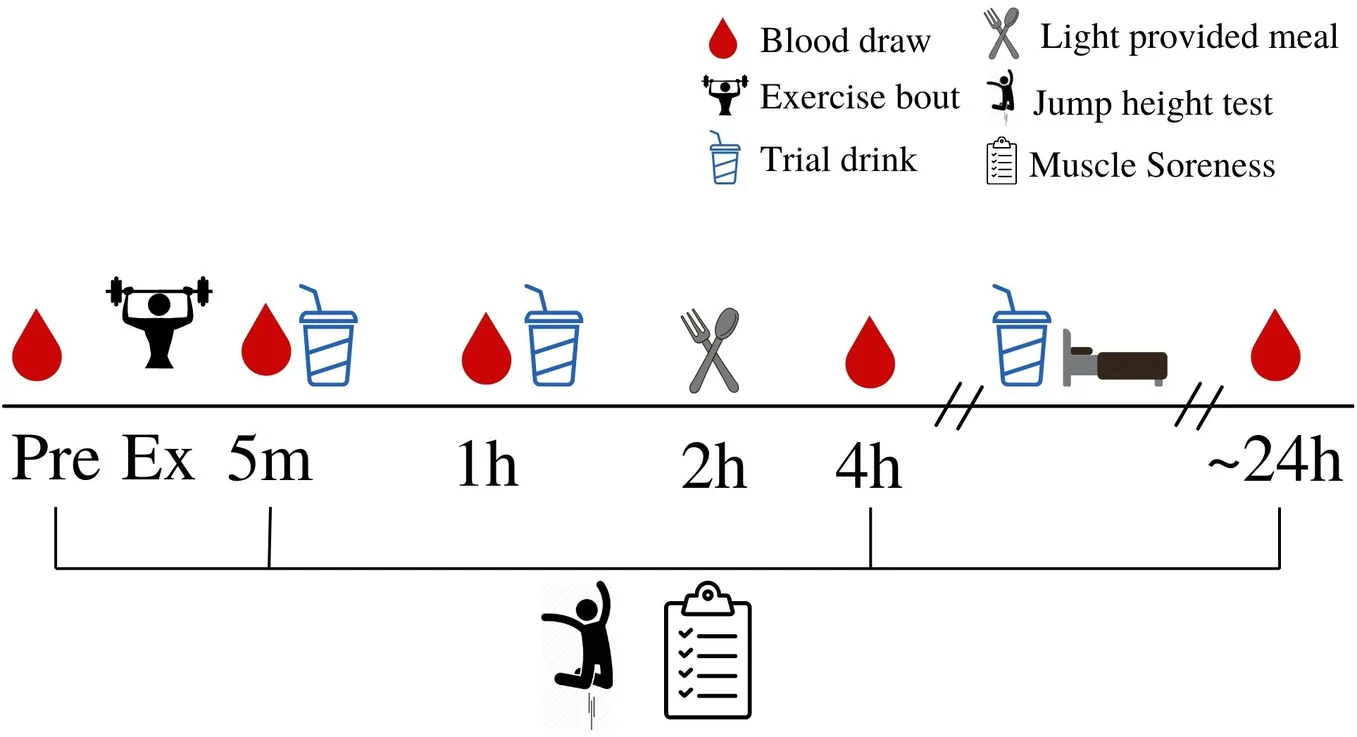
Detailed trial timeline depicting timing of all trial components. Each trial was identical with the exception of the trial supplement (carbohydrate, milk, Greek yogurt or water). Created using Canva (https://www.canva.com/).

Participants arrived at the laboratory between 0800 and 0900 h, following an overnight fast. Participants completed a trial readiness questionnaire to confirm they were fasted, rested (had not performed exercise), uncaffeinated, and were not experiencing any symptoms of illness that day or within the past week. A pre-exercise blood sample was performed, and pre-exercise muscle soreness and jump height were determined. Participants performed their high-intensity multi-modal exercise bout (Figure 1) in our lab gym facility, supervised by a personal trainer. Upon finishing their last cycling interval, participants returned to the laboratory as quickly as possible, and a post-exercise sample was taken (approximately 5 m post-exercise). Post-exercise muscle soreness and jump height were determined, and participants were provided with their first trial supplement. Upon finishing the supplement, a timer was started for the 1 h and 4 h blood samples. At 1 h, participants had a blood sample and a second trial supplement. At 2 h post-exercise, participants were provided a standardized meal (additional details on the standardized diet are provided below). At 4 h, a blood sample was taken, and muscle soreness and jump height were determined. Participants were then permitted to leave the lab and were provided with standardized meals for the remainder of the day and a third supplement. Participants were instructed to consume their food, refrain from treating their muscle soreness (no stretching, ice, heat, or exercise), and to return to the lab for their 24 h post-exercise sample (Figure 1).

### 2.5 Exercise protocol

All four trials consisted of an identical high-intensity multimodal exercise bout comprising plyometrics, resistance exercise and high-intensity interval cycling. All exercise sessions were supervised by a certified personal trainer. Each participant performed 3 sets of 10 jumps for each plyometric exercise (depth jumps, tuck jumps, split jumps, and seated jumps). Following plyometric exercises, participants performed all three resistance exercises (leg press, chest press, seated row) comprising 3 sets×10 repetitions at ∼70% e1RM. Each exercise was performed using a full range of motion, and verbal encouragement was provided to motivate the participant. A maximum of 2 min rest period was provided between sets and exercises. RPE was recorded following each set. Lastly, participants performed a high-intensity interval cycling protocol, consisting of a 4-minute warm-up, with at least 2 min cycling at 50% of their PPO. Participants performed 3 one-minute intervals at 90% of their PPO, with one minute of recovery at 50% of PPO between each interval. Heart rate was recorded in the last 10 s of each interval. During their first exercise session, if heart rate did not reach an average (over the 3 working intervals) of ≥85% of their estimated HR maximum, a fourth interval was required. The number of intervals was kept constant between trials, with participants performing 3 or 4 (for three participants) sets in each trial.

### 2.6 Supplement protocol

Supplements were consumed three times during the trial: post-exercise, 1 h post-exercise and 1 h before bed. A breakdown of trial supplements (CHO, MILK, GY, and WATER) can be seen in Table 1. The carbohydrate supplement (CHO) was made in-house by mixing 48 g of maltodextrin with water. For palatability, participants were offered the option of adding a sugar-free, calorie-free flavour enhancer (MiO Liquid Water Enhancer, Kraft Heinz, Chicago, USA or Crystal Light, Kraft Heinz, Chicago, USA; variety of flavours based on participant preference). Participants were provided with a pre-measured container of maltodextrin to take home and instructed on how to mix it with water to create their third supplement (consumed before bed). For the MILK arm, participants were provided 0% milk fat, Lactantia milk (Lactalis Group, Laval, France). For the GY arm, participants were provided Oikos 0% milk fat, high-protein, vanilla GY (Oikos, Danone, Paris, France). Participants were provided with pre-measured boluses of the milk and GY to consume at home for their third supplement (before bed). For the WATER arm, participants were provided with filtered tap water and were asked to measure out 500 mL of water at home to drink and consume as their third supplement (before bed). Participants were allowed to use the same sugar-free, calorie-free flavour enhancers for the water supplement, if desired. MILK, CHO and WATER were isovolumetric (500 mL), while CHO and MILK were matched for energy, and MILK and GY were roughly matched for protein. While participants and research personnel could not be blinded to the supplements, participants were blinded to our hypothesis and were blinded to their supplement allocation during the exercise session (i.e., supplement allocation was revealed only at the time of the first supplement, 5 m post-exercise).

**Table 1:** Nutritional breakdown of one bolus of each trial supplement.

Nutrients	CHO	MILK	GY	WATER
Calories (kcal)	180	180	149	0
Carbohydrate (g)	45	26	17	0
Protein (g)	0	18	19	0
Fat (g)	0	0.4	0	0
Calcium (mg)	0	600	257	0
Vitamin D (*μ*g)	0	10	0	0

CHO, carbohydrate; GY, Greek yogurt.

### 2.7 Muscle soreness

Participants were familiarized with a 0–5-point scale, with descriptive qualifiers [adapted from High et al., 1989 (69); Table 2] to rate their muscle tiredness/soreness/pain during the familiarization session. Participants were asked to rate their whole-body muscle soreness at pre-exercise, 5 m, 4 h, and 24 h post-exercise (Figure 1).

**Table 2:** Muscle soreness scale.

Rating	Description
0	No soreness.
1	Light soreness–soreness felt only when touched, a vague ache, does not affect movement.
2	Moderate soreness–Felt during touch or minor pain with activity (ex. walking up and down stairs), movement is slightly affected.
3	Moderate soreness and stiffness–Pain during touch and movement, increased stiffness with normal tasks and activity (ex. walking, jogging), movement is affected.
4	Intense pain, stiffness or weakness–pain, stiffness and weakness during normal activities (ex. walking, jogging), movement is greatly impacted.
5	Severe pain, stiffness, weakness–limited ability to move or perform activity.

Adapted from High et al., 1989 (69).

### 2.8 Jump height

During familiarization, participants were shown proper form for a standing vertical jump performance test (Tandem Sport, Louisville, KY, USA). Standing reach was measured at pre-exercise by instructing participants to stretch their dominant hand upwards as far as possible, and to walk through the movable veins of the vertical jump challenger. This was repeated 1–2 times to ensure accurate recording of their standing reach. Participants were instructed to swing their arms and bend their knees to engage in an explosive standing countermovement jump. Participants performed a minimum of 3 jumps or until performance plateaued. The maximal jump height achieved was recorded, and standing reach was subtracted to determine their jump height. Participants performed jump height testing pre-exercise, 5 m, 4 h, and 24 h post-exercise (Figure 1). At each timepoint, the participant was encouraged verbally to achieve their maximal performance.

### 2.9 Standardized diet and dietary records

On the day prior to the trial, participants were required to complete a dietary food log using the mobile application “Keenoa” (Keenoa, Montréal, QC, Canada), which is an image-based food logging system. Participants took photos of each meal and/or snack and uploaded these images into the application, where they were prompted to answer questions about meal composition and serving size. Participants were asked to replicate the same meals before each trial. During trial days, individualized, standardized, commercially available meals were provided to participants based on their estimated total daily energy expenditure. Total daily energy expenditure was estimated using the Harris-Benedict equation with a “lightly active” activity factor (70). All participants consumed the same vegetarian, low-protein lunch (vegetable noodle stir-fry, banana, and strawberry cereal bar), regardless of trial and total daily energy expenditure. Snacks (apple, banana, hummus and crackers, and strawberry cereal bar) and dinner meals (either balsamic chicken pasta meal or falafel bowl for vegetarian/halal participants) were provided and customized per/participant. Participants were allowed to consume these items based on their own food-timing preferences, provided they complied with the overnight fast preceding the 24 h blood sample. The exception was a protein bar (10 g of protein), which was provided to participants after the 4 h post-exercise testing period on the CHO and WATER arms only, as these arms provided less total daily protein than the dairy product arms. Trial meals were adjusted between arms to roughly match calorie intake (e.g., on the water arm, as this supplement contained no calories, participants were provided an extra dinner meal). Participants were also required to log these items on Keenoa and bring back any uneaten items to corroborate food records.

### 2.10 Blood sampling

Blood samples were taken pre-exercise, 5 m, 1 h, 4 h, and 24 h post-exercise from a vein in the antecubital fossa using a standardized venipuncture technique by a certified phlebotomist (E. Fraschetti). Approximately 22 mL of blood was collected at each timepoint, comprising one 6 mL red top vacutainer (serum), three 4 mL green top vacutainers (sodium heparin), and one 4 mL purple top vacutainer (EDTA). Green (NaHep) and purple (EDTA) top vacutainers (for plasma) were centrifuged (Rotina 38R, Hettich, Tuttlingen, Germany) immediately at 1,300 g for 10 min at 4 °C. Upon centrifugation, plasma was aliquoted into 1.5 mL Eppendorf tubes and stored at −80 °C for inflammatory cytokine analysis. Plasma (NaHep) concentrations of IL-1β, IL-1ra, IL-6, IL-8, IL-10, IFN-*γ*, and TNF were analyzed using automated ELISAs (Ella™, Protein Simple, Bio-Techne, MN, USA). IL-1β was undetectable for all participants, for all trials, at all timepoints. Intra-assay coefficients of variation were**:** IL-1ra: 2.37%; IL-6: 1.80%; IL-8: 1.87; IL-10: 3.83%; IFN-*γ*: 2.53%; TNF: 2.14%.

### 2.11 Hematocrit assessment

To account for changes in plasma volume following exercise and/or due to hydration status, hematocrit was measured at pre-exercise, 5 m, 1 h, and 4 h post-exercise. Hematocrit was measured from a portion of blood (300 µL) taken from the purple top EDTA vacutainer, in triplicate by the same investigator using microhematocrit capillary tubes (Fisherbrand™, 22–362566). Tubes were sealed with putty and spun in a microhematocrit centrifuge (Haematokrit 210, Hettich, Tuttlingen, Germany) at 10,000 RPM for 5 min. Following centrifugation, hematocrit was measured using an evaluation disk. All three values were recorded and averaged for the final hematocrit measure at each timepoint. If hematocrit values differed by >2%, that replicate was removed from the analysis (*n* = 12/320). At 24 h post-exercise, participants were assumed to have a similar hematocrit to pre-exercise. The percent plasma volume change was assessed for each timepoint relative to the pre-exercise value using the Van Beaumont formula (71).

### 2.12 Statistical analysis

Blood samples were unable to be obtained in two instances across all trials, once for one participant at 5 m post-exercise on CHO and another participant at 4 h on WATER. Hematocrit was unable to be measured for 6 timepoints due to equipment issues. Jump height was not collected for 5 timepoints, and muscle soreness was not collected at 3 timepoints. For one participant on their MILK trial only, cytokine concentrations were elevated. When compared to the average pre-exercise values across the other three trials, the MILK baseline values were higher by 6.3-fold for IL-10, 4.0-fold for IL-1ra, 20.0-fold for IFN-*γ*, 4.1-fold for TNF-α, 4.2-fold for IL-6, and 1.4-fold for IL-8. This indicates the participant was likely sick (without symptoms) during the trial. This entire arm was removed from the data set. All missing data were dealt with within the linear mixed model using restricted maximum likelihood estimation.

Prior to analysis, cytokine data were examined for outliers, and values >±2 SDs from the mean (*n* = 116, representing 4.9% of the 2,358 total datapoints) were replaced with the 2SD upper/lower limits, respectively. Following outlier treatment, data were assessed for normality using visual inspection and skewness and kurtosis z-scores (>3). All markers were deemed not normally distributed and log transformations improved normality for IL-10, IL-1ra, IFN-*γ*, IL-6, IL-8, and the ratio of TNF/IL-10. Log-transformed data were used in the linear mixed models. TNF normality was not improved with log transformation; however, skewness and kurtosis values were borderline (z-scores below 4), so non-transformed data were used in the model. Linear mixed models were conducted on the absolute plasma concentration (adjusted for the change in plasma volume) of each cytokine using R (R Core Team, Vienna, Austria, version 4.5.1) for Mac OS, using the lme4, lmerTest, and emmeans packages. Main fixed effects of trial (CHO, MILK, GY, and WATER), time (pre-exercise, 5 m, 1 h, 4 h, and 24 h post-exercise), and sex (male or female) and their interactions (trial*time, trial*sex, time*sex, and trial*time*sex) and a random effect of participant ID were added into the model. Trial order was included in the model as a covariate. To ensure that between-trial comparisons (main effect of trial, Trial|Time contrasts, and Trial|Sex contrasts) were not confounded by differences at pre-exercise, a second adjusted model that controlled for pre-exercise values and included only post-exercise timepoints (5 m, 1 h, 4 h and 24 h) was conducted. *p*-values from this adjusted model were reported for between-trial *post-hoc* pairwise comparisons, when a main effect or interaction was observed in the primary model.

One-way ANOVAs were conducted on the dietary intakes (energy intake, macronutrient and micronutrient intake) for the day of the trial. A one-way ANOVA was conducted on the exercise volume load (number of impacts, resistance exercise volume and RPE, interval cycling volume, RPE and % heart rate max). Linear mixed models were conducted for hematocrit, change in jump height and muscle soreness using fixed effects of trial (CHO, MILK, GY, and WATER) and time (pre-exercise, 5 m, 1 h, 4 h for hematocrit; 5 m, 4 h, 24 h for change in jump height, and pre-exercise, 5 m, 4 h, 24 h for muscle soreness).

Following a significant *F*-test, *post hoc* analyses were conducted and corrected using the Benjamini-Hochberg false discovery rate. Significance was set to *p* ≤ 0.05 for all tests. The figures were made using Prism 10.2.3 for MacOS (GraphPad software, San Diego, CA, USA). Values are presented as mean ± standard error (SE) for Figures and mean ± standard deviation (SD) for Tables.

## 3 Results

### 3.1 Participant characteristics

Baseline participant characteristics measured at the familiarization session are outlined in Table 3.

**Table 3:** Participant characteristics during familiarization (*n* = 20).

Characteristics	Mean ± SD
Sex (F/M)	12/8
Age (years)	22.7 ± 4.0
Height (cm)	165.5 ± 8.8
Weight (kg)	60.3 ± 8.3
BMI (kg/m^2^)	22.0 ± 2.3
Body Fat (%)	23.4 ± 9.6
PPO (W)	166 ± 41

BMI, body mass index; W, watts; PPO, peak power output. Values are mean ± SD.

### 3.2 Exercise data

There were no differences between trials for the number of impacts, percent e1RM, volume load or RPE for resistance exercise and interval cycling, nor a difference between trials for the percent of heart rate max achieved during cycling (Table 4).

**Table 4:** Comparison of the number of impacts, resistance exercise volume load, and cycling volume load for all trials (*n* = 20).

Exercises	CHO	MILK	GY	WATER	*p*-value
Impacts	120	120	120	120	1.000
Chest Press					
%e1RM	69.1 ± 4.6	68.4 ± 4.9	69.1 ± 4.6	69.7 ± 4.0	0.272
Volume Load (kg)	1075 ± 460	1066 ± 458	1075 ± 460	1080 ± 454	0.485
RPE	4.7 ± 1.5	5.0 ± 1.2	5.3 ± 1.3	4.6 ± 1.5	0.109
Leg Press					
%e1RM %	68.3 ± 8.7	68.3 ± 8.7	68.3 ± 8.7	68.3 ± 8.7	0.330
Volume Load (kg)	2190 ± 1097	2190 ± 1097	2189 ± 1096	2190 ± 1097	0.330
RPE	5.3 ± 1.8	5.5 ± 1.3	5.5 ± 1.7	5.1 ± 1.8	0.580
Seated Row					
% e1RM	67.6 ± 5.9	67.6 ± 5.9	67.6 ± 5.9	67.6 ± 5.9	1.000
Volume Load (kg)	939 ± 320	939 ± 320	939 ± 320	939 ± 319	1.000
RPE	5.4 ± 1.7	5.6 ± 1.7	5.8 ± 1.4	5.5 ± 1.8	0.448
Cycling					
% HR max	89.5 ± 8.0	92.3 ± 5.3	92.9 ± 3.9	92.5 ± 6.5	0.078
Volume Load (kJ)	28.4 ± 7.1	28.2 ± 7.0	27.6 ± 6.9	28.1 ± 7.3	0.277
RPE	7.5 ± 2.1	7.8 ± 1.9	8.1 ± 1.7	7.6 ± 1.7	0.292

Volume load was determined by sets*repetitions*weight for resistance exercises and sets*time*Watts for cycling. RPE was measured using Borg’s 0–10 Critical Rating scale (66). %HRmax and cycling RPE were taken from the last working interval (third or fourth). HRmax was estimated using 220-age (68). CHO, carbohydrate; GY, Greek yogurt; %e1RM, percentage of estimated one repetition maximum; RPE, ratings of perceived exertion; %HRmax, percentage of heart rate maximum. Values are mean ± SD.

### 3.3 Dietary analysis

There were no differences between trial arms in energy intake, macro- or micronutrient intake on the day prior to the trial (*p* > 0.05, for all), except for sodium (Table 5). Sodium intake was higher on MILK and GY vs. CHO (*p* < 0.05, for both), and higher on GY vs. WATER (*p* = 0.035). There were no differences in energy intakes on the day of the trial (Table 6). There were significant differences in daily protein, fat (including saturated fat), and carbohydrate (including sugar and fibre) intakes between trials, primarily reflecting the supplements consumed (i.e., by design).

**Table 5:** Comparison of dietary intake for the day before the trial (*n* = 20).

Dietary Variable	CHO	MILK	GY	WATER	*p*-value
Energy intake (kcal)	1533 ± 544	1620 ± 633	1498 ± 519	1443 ± 543	0.369
Protein (g)	64 ± 29	68 ± 30	65 ± 31	58 ± 24	0.460
Fat (g)	57 ± 28	61 ± 34	59 ± 30	55 ± 28	0.803
Saturated Fat (g)	17 ± 9	20 ± 12	21 ± 12	18 ± 12	0.387
Trans Fat (g)	0 ± 0	1 ± 1	1 ± 1	1 ± 0	0.697
Carbohydrate (g)	194 ± 67	203 ± 77	182 ± 59	182 ± 68	0.243
Sugar (g)	66 ± 35	75 ± 49	62 ± 30	72 ± 35	0.281
Fibre (g)	11 ± 6	12 ± 6	12 ± 5	11 ± 5	0.701
Calcium (mg)	437 ± 275	536 ± 348	536 ± 400	452 ± 220	0.349
Iron (mg)	9 ± 5	9 ± 5	9 ± 5	9 ± 5	0.873
Sodium (mg)	2049 ± 884^a^	2659 ± 1453^bc^	2697 ± 1479^b^	2190 ± 1181^ac^	**0.036**
Potassium (mg)	1569 ± 711	1697 ± 731	1586 ± 789	1694 ± 743	0.678
Vitamin D (IU)	98 ± 130	129 ± 146	119 ± 134	134 ± 147	0.331
Magnesium (mg)	166 ± 92	172 ± 100	168 ± 99	177 ± 86	0.895
Phosphorus (mg)	699 ± 381	738 ± 418	735 ± 479	673 ± 308	0.746

Differing letters represent differences between trials. CHO, carbohydrate; GY, Greek yogurt. *p*-values <0.05 are bolded. Values are mean ± SD.

**Table 6:** Comparison of dietary intake for the day of the trial (i.e., the day of exercise; *n* = 20).

Dietary Variable	CHO	MILK	GY	WATER	*p*-value
Energy intake (kcal)	2017±237	1969±262	1979±214	1975±242	0.231
Protein (g)	58±10^a^	101±14^b^	107±9^c^	78±12^d^	**<0.001**
Fat (g)	35±7^a^	34±7^b^	33±7^ab^	46±7^c^	**<0.001**
Saturated Fat (g)	7±1^a^	5±1^b^	5±1^c^	8±1^d^	**<0.001**
Trans Fat (g)	0±0	0±0	0±0	0±0	0.109
Carbohydrate (g)	385±38^a^	332±39^b^	333±33^b^	334±39^b^	**<0.001**
Sugar (g)	88±14^a^	161±17^b^	149±13^c^	111±18^d^	**<0.001**
Fibre (g)	27±4^a^	28±5^b^	31±5^c^	35±6^d^	**<0.001**
Calcium (mg)	242±27^a^	1937±241^b^	988±30^c^	319±36^d^	**<0.001**
Iron (mg)	12±2^a^	11±2^bc^	11±2^c^	15±2^d^	**<0.001**
Sodium (mg)	1839±315^a^	2379±355^bd^	2045±291^c^	2343±313^d^	**<0.001**
Potassium (mg)	1981±365^a^	4511±548^b^	3280±280^c^	2622±426^d^	**<0.001**
Vitamin D (IU)	0±0^a^	1032±267^b^	0±0^a^	0±0^a^	**<0.001**
Magnesium (mg)	64 ± 15^ad^	234 ± 25^b^	128 ± 4^c^	67 ± 16^d^	**<0.001**
Phosphorus (mg)	63 ± 14^ad^	1537 ± 210^b^	939 ± 9^c^	66 ± 14^d^	**<0.001**

Differing letters represent differences between trials. CHO, carbohydrate; GY, Greek yogurt. *p*-values <0.05 are bolded. Values are mean ± SD.

### 3.4 Jump height and muscle soreness

**Figure 2:**
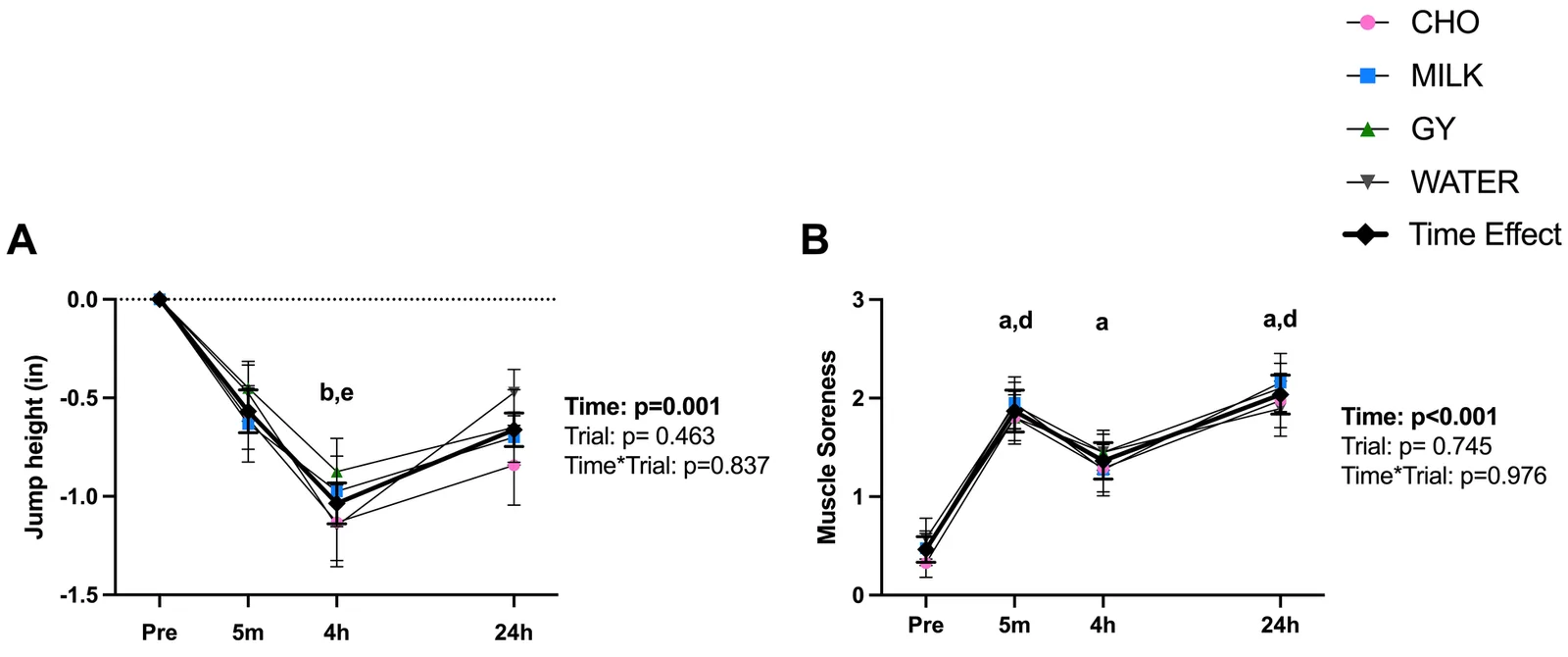
Change in jump height **(A)** and muscle soreness ratings **(B)** over time [pre-exercise (pre), 5 m, 4 h, and 24 h post-exercise] for all arms. The collapsed effect of time (all groups) is denoted in black and letters denote a difference from pre-exercise (a), 5 m (b), 4 h (d) and 24 h (e) Values are mean ± SE.

There was a main effect of time for the change in jump height, whereby jump height was lower at 4 h vs. 5 m (*p* = 0.001) and 24 h (*p* = 0.003) (Figure 2A). There was a main effect of time for muscle soreness, whereby soreness increased at all timepoints vs. pre-exercise (*p* < 0.001, for all), and soreness was higher at 5 m and 24 h vs. 4 h (*p* < 0.001, for both; Figure 2B).

### 3.5 Plasma cytokine concentrations

#### 3.5.1 Pro-inflammatory cytokines

**Figure 3:**
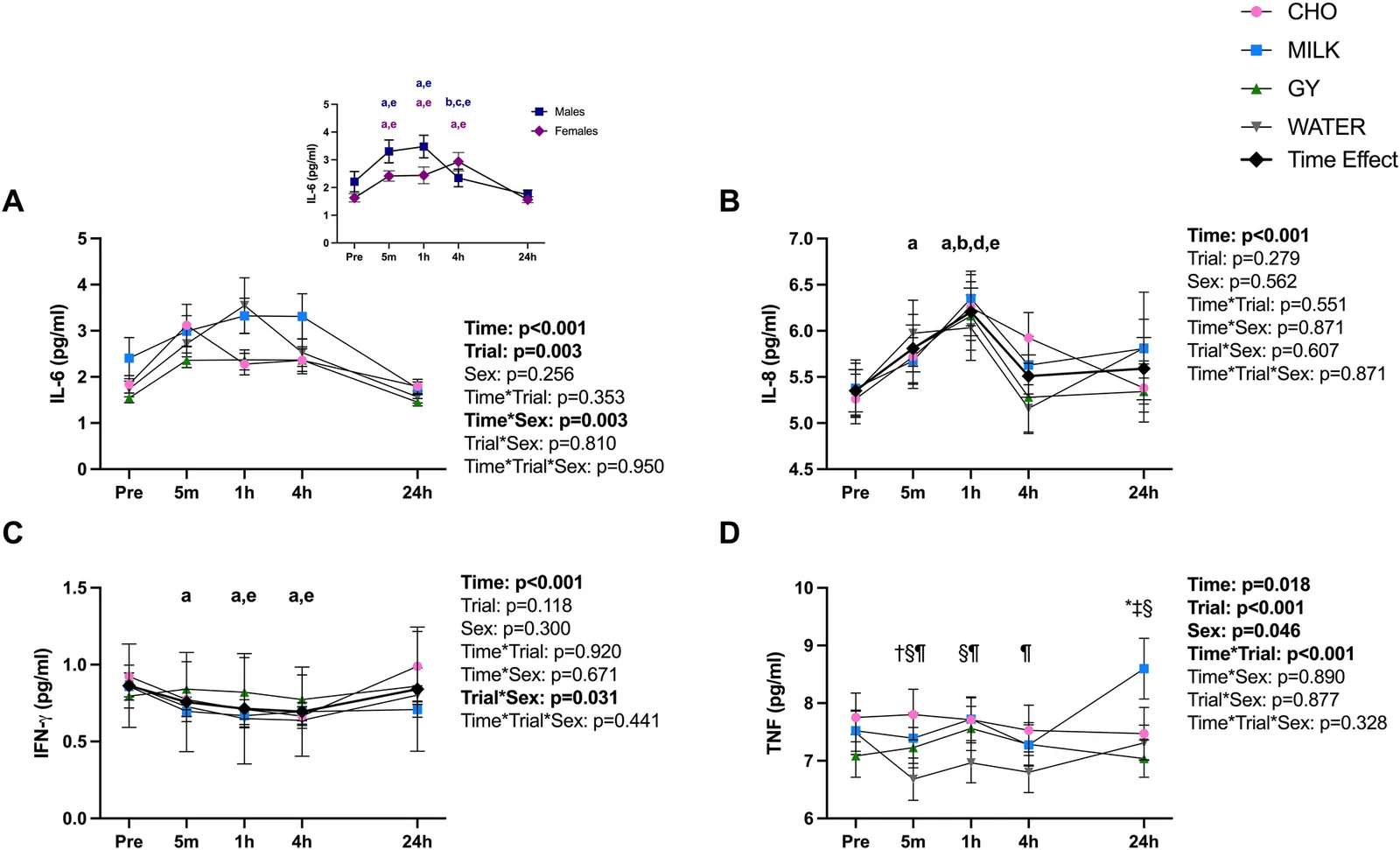
Concentration of interleukin (IL)-6 (**A** and inset), IL-8 **(B)**, interferon-*γ* (IFN-*γ*; **C**), and tumor necrosis factor (TNF; **D**) over time [pre-exercise (pre), 5 m, 1 h, 4 h, and 24 h post-exercise] for all trial arms. Following a time effect, without an interaction, the collapsed effect of time (all groups) is denoted in black, and letters denote a difference between timepoints, a difference from pre-exercise (a), 5 m (b), 1 h (c), 4 h (d) and 24 h (e) Following a time*sex interaction, the collapsed effect of trial for males (dark blue) and females (dark purple) is indicated in the inset **(A)**, with the colours of the symbols corresponding to males (dark blue) and females (dark purple). Symbols denote differences between arms: * = CHO vs. MILK, † = CHO vs. WATER, ‡ = MILK vs. GY, § = MILK vs. WATER, ¶ = GY vs. WATER. Values are mean ± SE.

There was a time-by-sex interaction for IL-6 (Figure 3A). In females, IL-6 was elevated at 5 m, 1 h, and 4 h vs. pre-exercise and 24 h (*p* < 0.001, for all). Whereas in males, IL-6 was elevated at 5 m and 1 h only vs. pre-exercise and 24 h (*p* < 0.001, for all). Further, in males, the concentration at 4 h was lower than 5 m and 1 h (*p* < 0.001, for both) and higher than 24 h (*p* = 0.022). There was a main effect of trial, however, after controlling for pre-exercise values, *post-hoc* comparisons revealed no differences between trials. There was a main effect of time for IL-8 (Figure 3B), IL-8 increased at 5 m vs. pre-exercise (*p* = 0.014), peaking at 1 h [i.e., was higher than all other times, (*p* < 0.05, for all)]. There was a main effect of time and a trial-by-sex interaction for IFN-*γ* (Figure 3C). For the main effect of time, IFN-*γ* decreased at 5 m, 1 h, and 4 h vs. pre-exercise (*p* < 0.001, for all). At 24 h, IFN-*γ* was significantly higher than 1 h, and 4 h (*p* < 0.05, for all). The trial-by-sex interaction revealed no difference between trials for females or males after controlling for pre-exercise values. There was a main effect of sex and a time-by-trial interaction for TNF (Figure 3D). The main effect of sex revealed a greater concentration of TNF over the intervention in males vs. females. The time-by-trial interaction revealed differences between trials at several timepoints after controlling for pre-exercise values (Figure 3D). At 5 m WATER had lower TNF than all arms (*p* < 0.05, for all) and at 1 h WATER had lower TNF than MILK and GY (*p* = 0.034 and *p* = 0.010, respectively). At 4 h, WATER had lower TNF than GY (*p* = 0.037). At 24 h, MILK had higher TNF than all arms (*p* < 0.001, for all). The time-by-trial interaction also revealed differences in how the trials changed over time (Supplementary Table 1). There was no change in TNF over time in CHO or GY. In WATER, TNF decreased at 5 m and 4 h vs. pre-exercise (*p* < 0.05, for both). In MILK, TNF was higher at 24 h vs. all other timepoints (*p* < 0.01, for all). IL-1β was undetectable for all participants, for all trials, at all timepoints.

#### 3.5.2 Anti-inflammatory cytokines

**Figure 4:**
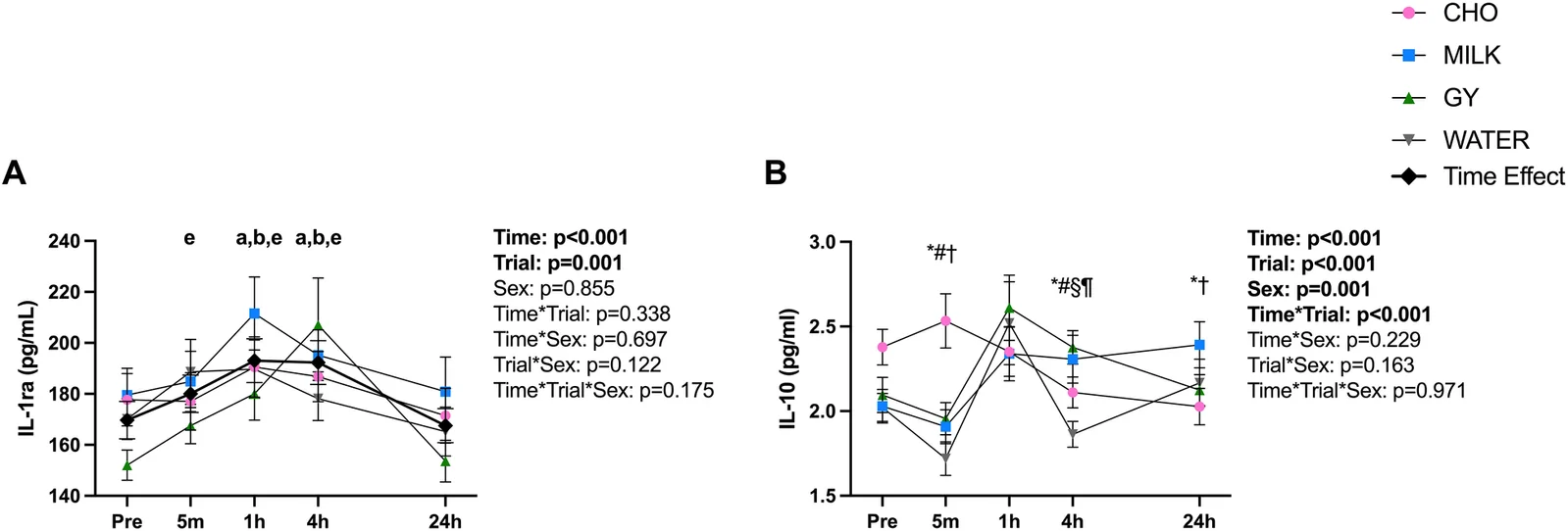
Concentration of interleukin (IL)-1 receptor antagonist **(A)** and IL-10 **(B)** over time [pre-exercise (pre), 5 m, 1 h, 4 h, and 24 h post-exercise] for all trial arms. Following a time effect, without an interaction, the collapsed effect of time (all groups) is denoted in black, and letters denote a difference between timepoints, a difference from pre-exercise (a), 5 m (b), 1 h (c), 4 h (d) and 24 h (e) Symbols denote differences between arms: * = CHO vs. MILK, # = CHO vs. GY, † = CHO vs. WATER, § = MILK vs. WATER, ¶ = GY vs. WATER. Values are mean ± SE.

For IL-1ra, there was a main effect of time (Figure 4A), IL-1ra was elevated at 1 h and 4 h compared to all other timepoints (*p* < 0.05, for all). The concentration returned to baseline by 24 h, which was lower than 5 m (*p* = 0.033). There was a main effect of trial, however, after controlling for pre-exercise values, *post-hoc* comparisons revealed no differences between trials. There was a main effect of sex and a time-by-trial interaction for IL-10 (Figure 4B). The main effect of sex revealed a greater concentration of IL-10 over the intervention in males vs. females. The time-by-trial interaction revealed differences between trials at several timepoints after controlling for pre-exercise values (Figure 4B). At 5 m, CHO had higher IL-10 than all other arms (*p* < 0.05, for all). At 4 h, the dairy arms (MILK and GY) had higher IL-10 vs. CHO and WATER (*p* < 0.05, for all). At 24 h, CHO had lower IL-10 than WATER and MILK (*p* = 0.043 and 0.001, respectively) The time-by-trial interaction also revealed differences in how the trials changed over time (Supplementary Table 1). For CHO, IL-10 was lower at 24 h vs. pre-exercise, 5 m, and 1 h (*p* < 0.05 for all), and IL-10 was lower at 4 h vs. 5 m (*p* = 0.032). For WATER, IL-10 was lower at 5 m vs. pre-exercise (*p* = 0.011), higher at 1 h vs. all other timepoints (*p* < 0.05 for all), and higher at 24 h vs. 4 h (*p* = 0.022). For GY, IL-10 was higher at 1 h vs. pre-exercise, 5 m and 24 h, and 4 h was higher vs. 5 m (*p* < 0.05 for all). For MILK, IL-10 was higher at 1 h, 4 h, and 24 h vs. 5 m (*p* < 0.01, for all), and IL-10 was higher at 24 h vs. pre-exercise (*p* = 0.030).

#### 3.5.3 TNF/IL-10 ratio

**Figure 5:**
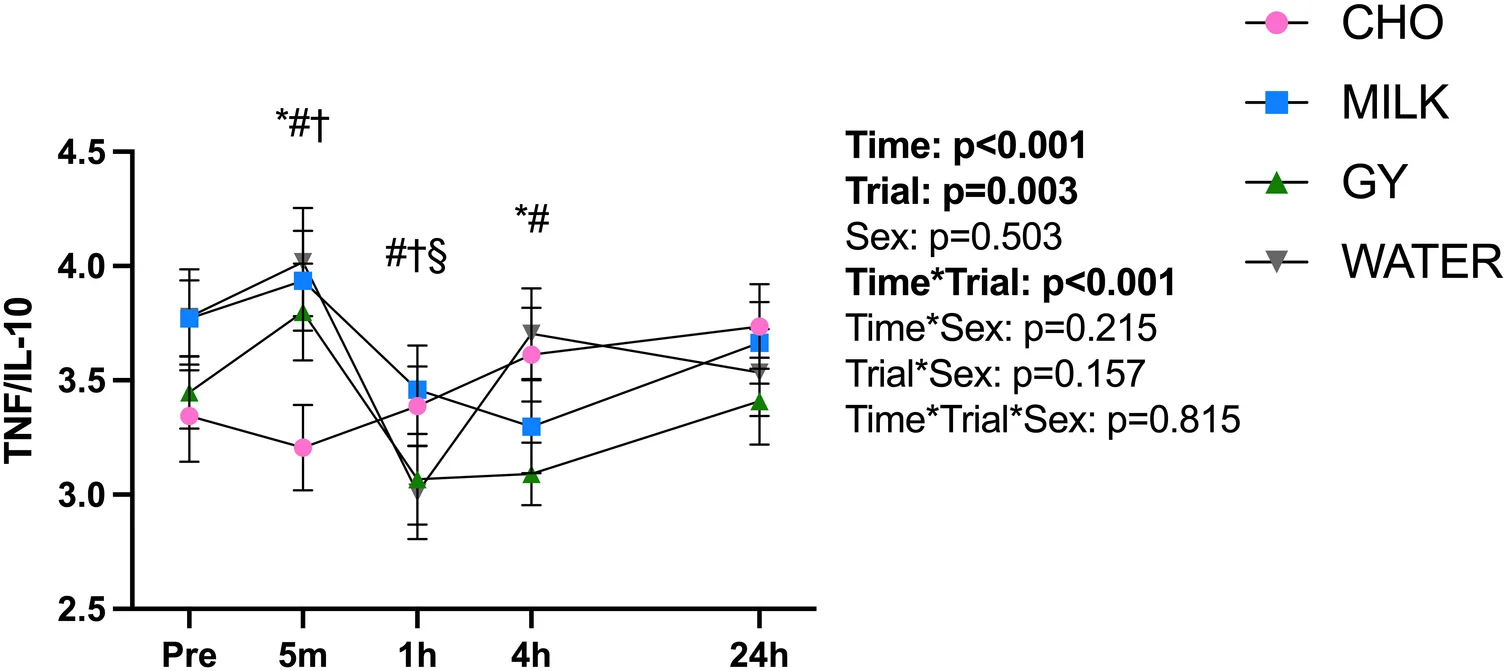
Concentration of tumor necrosis factor/interleukin-10 (TNF/IL-10) over time [pre-exercise (pre), 5 m, 1 h, 4 h, and 24 h post-exercise] for all trial arms. Symbols denote differences between arms: * = CHO vs. MILK, # = CHO vs. GY, † = CHO vs. WATER, § = MILK vs. WATER. Values are mean ± SE.

There was a main time-by-trial interaction for the ratio of TNF/IL-10 (Figure 5). The time-by-trial interaction revealed differences between trials at many timepoints after controlling for pre-exercise values (Figure 5). At 5 m, the ratio was lower in CHO vs. all other arms (*p* < 0.05, for all). At 1 h, the ratio was lower in WATER vs. CHO and MILK (*p* < 0.05, for both) and lower in GY vs. CHO (*p* = 0.050). At 4 h, the ratio was higher in CHO vs. the dairy arms (MILK and GY). The time-by-trial interaction also revealed differences in how the trials changed over time (Supplementary Table 1). For CHO, TNF/IL-10 was higher at 24 h vs. 5 m (*p* = 0.027). For WATER, TNF/IL-10 was lower at 1 h vs. all other times (*p* < 0.01 for all), and higher at 5 m vs. 24 h (*p* = 0.028). For GY, the ratio was lower at 1 h vs. pre-exercise (*p* = 0.035) and lower at 1 h and 4 h vs. 5 m (*p* < 0.001, for both). For MILK, the ratio was lower at 1 h vs. 5 m (*p* = 0.032), and 4 h was lower than pre-exercise, 5 m, and 24 h (*p* < 0.05 for all).

## 4 Discussion

Our study examined the influence of different post-exercise nutritional supplements on the exercise-induced inflammatory response on circulating cytokines. Irrespective of nutrition, we observed main effects of time for IL-1ra, IL-8 and IFN-*γ*, with increases in IL-8 and IL-1ra post-exercise, and decreases in IFN-*γ*. For IL-6, we observed a sex-dependent response, whereby IL-6 increased in both males and females at 5 m and 1 h, in all trial arms; however, in females, IL-6 remained elevated at 4 h. Together, these results illustrate that the exercise bout induced an inflammatory response. Following post-exercise MILK or GY consumption, there was a higher IL-10 concentration vs. CHO and WATER, and a lower TNF/IL-10 ratio vs. CHO at 4 h post-exercise. Furthermore, IL-10 decreased in CHO at 24 h (Supplementary Table 1) and was lower at 24 h vs. WATER and MILK. Together, these results illustrate an enhanced IL-10 response following post-exercise dairy (MILK or GY) consumption vs. CHO and WATER. Despite no differences between groups in muscle soreness or counter movement jump height (a measure of performance), the dairy arms elicited a more anti-inflammatory environment post-exercise, which may indicate an earlier onset of the anti-inflammatory phase of exercise-induced inflammation.

The circulating cytokine response to exercise, without nutrition, has been well characterized (1–6). In the present study, we observed increases in both IL-8 and IL-1ra following exercise, with no differences between our nutritional interventions. Increases in IL-8 (1, 5, 6) and IL-1ra (1, 4) following exercise are well documented within the literature. Studies investigating post-exercise nutrition have also observed increases in IL-1ra post-exercise with no differences between flavoured milk and carbohydrate controls (34, 72). Similarly, in a study by Ferguson-Stegall and colleagues, an increase in IL-8 was observed with post-exercise nutrition, with no differences between flavoured milk and carbohydrate or water controls (34). In contrast, other studies have observed no change in IL-8 following exercise; however, these studies were in trained individuals (72, 73), whereas ours and Ferguson-Stegall and colleagues (34) examined untrained individuals. Trained individuals have an attenuated inflammatory response following exercise compared to untrained individuals, where the stimulus exhibits greater novelty (2). We also observed a decrease in IFN-*γ* following exercise, which returned to baseline concentrations by 24 h post-exercise. A decrease in IFN-*γ* following exercise has been observed within the literature (4); however, it is not often investigated following post-exercise dairy consumption. IFN-*γ* is primarily produced by T cells and NK cells (6), which predominate later in the pro-inflammatory phase (5, 6). Early in the pro-inflammatory phase of exercise-induced inflammation, innate immune cells, such as neutrophils and monocytes/macrophages, dominate (5, 6), thus, there may be reductions in circulating adaptive immune cells (like T cells), leading to a reduction in mediators produced by these cells (e.g., IFN-*γ*). Examination of T cell populations, among other immune cells, following exercise would provide greater insight into exercise-induced inflammation and may aid in the interpretation of the cytokine responses.

An increase in IL-6 following exercise, as observed in the present study, has been well characterized in the literature (74, 75). Typically, an increase in IL-6 is observed during and/or immediately following exercise (74, 75). It is hypothesized that this increase in IL-6 is due to production of IL-6 at the level of the muscle, as IL-6 plays an important role in maintaining glucose availability during exercise (74, 75). This initial increase in IL-6 is often deemed to have anti-inflammatory action, due to the abundance of IL-6 receptors in the muscle, which promotes classical/anti-inflammatory IL-6 signalling (76–79). Following this initial increase in IL-6, there is often a secondary rise in IL-6, which is hypothesized to derive from immune cells and act in a pro-inflammatory manner (78, 79). Measurement of IL-6 production by immune cells may help elucidate the source of IL-6 following exercise, providing insight into the pro- and anti-inflammatory actions of this pluripotent cytokine. Interestingly, we observed a time-by-sex interaction for IL-6, whereby there was a more sustained IL-6 response (up to 4 h) in females, but in males, IL-6 concentration returned to baseline by 4 h post-exercise. While it is well known that there are key sex differences in immune function (80), little research has investigated sex-based differences (81). Future studies should continue to investigate sex-specific responses to exercise and how these may be differentially modulated following post-exercise nutrition.

Following MILK, there was an increase in TNF at 24 h post-exercise, which was greater than all other timepoints and all other trial arms (Supplementary Table 1). In our previous research, which followed a similar supplement protocol, we did not observe an increase in TNF 24 h post-exercise with milk consumption (36). However, in the present investigation, we provided one additional supplement to the participants, which was consumed before bed. As both nutritional quality and quantity may influence how an individual tolerates post-exercise nutrition (73), this difference in methodology may help explain the discrepancy in findings between our two studies. Nonetheless, while TNF was elevated following MILK at 24 h post-exercise, the TNF/IL-10 ratio revealed no difference at 24 h from baseline in MILK (Supplementary Table 1) nor was there a difference between trial arms at 24 h post-exercise. This indicates that the increase in TNF may be appropriately compensated for with a concomitant and proportional increase in IL-10. IL-10 can directly inhibit synthesis of many pro-inflammatory mediators, including TNF (3). We have previously demonstrated that an increase in TNF was associated with an increase in IL-10 following exercise training (82). Nonetheless, this increase in TNF following MILK may warrant further investigation. TNF was also lower in WATER at 5 m vs. all other trials, lower at 1 h vs. GY and MILK, and lower at 4 h vs. GY. This was likely driven by the reduction of TNF between pre-exercise and 5 m in the WATER trial, where there was no change in TNF between these timepoints for the other trials (Supplementary Table 1). As nutrition did not influence this 5 m timepoint, this difference cannot be attributed to our nutritional intervention and may relate to confounding factors that we were unable to control within the intervention (e.g., weather, sleep quality and/or stress levels).

IL-10 was higher at 4 h post-exercise in MILK and GY vs. both CHO and WATER. Further, the TNF/IL-10 ratio was lower (less inflammatory) in the dairy arms, MILK and GY, vs. CHO at 4 h post-exercise. Despite differences in wholefood matrices, fermentation, and digestion/absorption kinetics between milk and GY, both dairy arms acted similarly, contrary to our hypothesis, however, MILK had elevated TNF at 24 h vs. pre-exercise, which was not observed in GY. Importantly, both milk and GY exhibited a decrease in the TNF/IL-10 ratio at 4 h, and there was no difference between trials for the TNF/IL-10 ratio at 24 h post-exercise. Thus, GY may induce a greater anti-inflammatory environment over the 24 h post-exercise period, compared to MILK when assessing cytokines separately. While the increases in IL-10 and changes in the TNF/IL-10 ratio were small, these findings are consistent with previous literature (52, 53). For example, higher IL-10 following whey protein consumption has also been previously observed compared to water (52) and carbohydrate controls (53). Following a high-intensity interval swimming protocol, adolescent athletes were provided either water, whey protein isolate, or an isoenergetic carbohydrate beverage (52). Following whey protein consumption, there was no difference between groups for TNF or IL-6, but IL-10 was higher in the whey protein group vs. the water group 8 h post-exercise (52). Similarly, Kerasioti et al. provided participants with a carbohydrate/whey protein bar, or a carbohydrate-only bar, following an exhaustive cycling protocol, observing greater increases in C-reactive protein and IL-6 at 4 h post-exercise following CHO, and a greater increase in IL-10 following carbohydrate and whey protein bar (53). Together with the present study, these studies illustrate the ability of post-exercise whey and dairy consumption to modulate and increase the anti-inflammatory cytokine IL-10 (52, 53). This increase in IL-10 in the mid-phase of recovery from exercise may illustrate a switching of macrophage phenotype from M1-like (pro-inflammatory) to M2-like (anti-inflammatory) to promote muscle repair, as IL-10 is a key cytokine responsible for the onset of the anti-inflammatory phase of the exercise-induced inflammatory response (5, 6). This may correspond to an improvement in recovery time, however, we observed no differences between our nutritional interventions for both muscle soreness and exercise performance (countermovement jump height). Importantly, these subjective outcome measurements may not have been sensitive enough to capture the subtle influence of nutrition. Future research should consider physiological measurements (i.e., blood markers of muscle damage) and subjective measures with greater sensitivity and more points of discrimination (i.e., granularity). While it is speculative that the increase in IL-10 would translate to improved recovery, elevated IL-10 following dairy/dairy protein consumption does appear to be a consistent finding across different studies (52, 53). It may be prudent for future studies to also investigate immune cell responses and/or obtain muscle biopsies following exercise and supplement consumption to gain a greater understanding of the degree of muscle damage, source of cytokine production, and the local (i.e., muscle) inflammatory response. Furthermore, examination of more post-exercise timepoints between 4 and 24 h post-exercise may allow for greater understanding of the IL-10 response, including when its concentration peaks following post-exercise dairy consumption.

While the dairy arms had an increase in IL-10 (and concomitant decrease in the TNF/IL-10 ratio) over the intervention, the CHO arm displayed a decrease in IL-10, whereby IL-10 was lower at 24 h post-exercise vs. WATER and MILK (Supplementary Table 1). While carbohydrates are commonly consumed post-exercise to support glycogen resynthesis (83), from an inflammatory standpoint, these supplements may not favourably affect the pro- and anti-inflammatory responses to exercise. This may relate to their high glycemic index and subsequent hyperglycemia, which has been shown to induce a transient inflammatory response (63). It is important to note that IL-10 was higher in CHO at 5 m vs. all other trials, however, as stated previously, nutrition did not influence this 5 m timepoint (i.e., this sampling timepoint was prior to the ingestion of any post-exercise nutrition), thus, this difference is not mediated by the nutritional intervention. Interestingly, there was an increase in IL-10 at 1 h post-exercise following WATER, which translated to a lower TNF/IL-10 ratio at 1 h post-exercise that was lower than all other timepoints and lower than CHO and MILK (Supplementary Table 1). This may represent a transient benefit of acute fasting, as decreases in pro-inflammatory cytokines have been observed with fasting (84). In the current investigation, participants in the water arm arrived at the lab fasted and remained fasted for 13–14 h (until eating their standardized lunch at 2 h post-exercise). Because high-intensity exercise increases energy demand, exercise may enhance the benefits of fasting relative to resting conditions (85). However, while some individuals prefer to exercise in a fasted state, it may not be practical to recommend that individuals remain fasted following exercise to decrease inflammation. Additionally, this benefit appears transient as it disappears following consumption of the standardized lunch meal.

This study has several strengths. First, the use of a crossover design where each participant serves as their own control helps reduce interparticipant variability, which can be high when investigating inflammatory markers. We also controlled dietary intakes for the exercise trial day, providing participants with lunch, snacks, and dinner for the entire day. This allowed us to ensure that, from a nutrition standpoint, there were limited differences between trials except for the post-exercise supplementation. Furthermore, in controlling for the diet on the day of exercise, we provided the non-dairy arms (CHO and WATER) with a protein bar to ensure protein was adequate despite having a non-protein supplement (average protein intake was 0.97 g/kg/day and 1.35 g/kg/day for CHO and WATER, respectively). This study also has several limitations. While we observed benefits for the dairy arms in promoting an anti-inflammatory environment post-exercise, this did not translate to a benefit for muscle soreness or performance (countermovement jump height). While we may have been underpowered for the secondary analyses of muscle soreness and jump height, our null findings may also be explained by limitations associated with the measurement approaches used to assess these outcomes. For muscle soreness, we used a 5-point descriptive scale; however, a visual analog scale may provide greater sensitivity and more granularity, although still subjective. Similarly, our vertical jump height apparatus measured only to the nearest inch, which is a large difference in performance. Use of a different vertical jump height apparatus, allowing for smaller intervals (0.5 inch), or the implementation of other, more sensitive performance measures may allow us to potentially capture smaller effects. Also, although we corrected for multiple comparisons, some *p*-values were close to the significance threshold (*p*≤0.05), which may increase the risk of type 1 error. Lastly, the use of wholefood dairy products limits our ability to determine whether the observed effects were attributable to specific dairy components (e.g., whey protein) or to the dairy food matrix as a whole. Consequently, the mechanisms underlying the observed responses remain unclear. Future research should compare isolated dairy components with wholefood dairy products to better elucidate the contribution of the dairy matrix to post-exercise inflammatory outcomes.

Overall, we observed a modest benefit of post-exercise dairy consumption with minimal differences between the dairy arms in modulating post-exercise inflammation. Specifically, there was higher IL-10 4 h post-exercise in MILK and GY vs. CHO and WATER, which translated to a lower/less inflammatory TNF/IL-10 ratio at 4 h in the dairy arms (vs. CHO). Although TNF concentrations were elevated 24 h post-exercise following MILK consumption, no differences were observed between treatment arms for the TNF/IL-10 ratio. This suggests that the increase in TNF may have been counterbalanced by a concomitant increase in IL-10, maintaining the overall balance between pro- and anti-inflammatory responses. Nevertheless, the elevated TNF concentration following MILK warrants further investigation. Collectively, these findings suggest that post-exercise dairy consumption, particularly GY, may contribute to a more anti-inflammatory post-exercise environment. This could facilitate an earlier onset of the anti-inflammatory phase of the exercise-induced inflammatory response, which could be associated with improved recovery.

## Data Availability

The raw data supporting the conclusions of this article will be made available by the authors, without undue reservation.
