# Supplementary material for: Post-exercise consumption of milk or Greek yogurt may support the anti-inflammatory environment following high-intensity exercise
Source: Front Sports Act Living. 2026 Aug 26;8:1911130. doi: 10.3389/fspor.2026.1911130 (PMC13556160; doi:10.3389/fspor.2026.1911130)
Supplement: Supplementary file 1 [file Table1.docx]

Supplementary Material

**Supplementary Table 1:** Absolute concentrations of systemic cytokines with between-trial differences and within-trial differences across time.

| Cytokine (pg/ml) | Sex | CHO | | | | | MILK | | | | | GY | | | | | WATER | | | | | Time | Trial | Time*Trial | Time*Sex |
| --- | --- | --- | --- | --- | --- | --- | --- | --- | --- | --- | --- | --- | --- | --- | --- | --- | --- | --- | --- | --- | --- | --- | --- | --- | --- |
|  |  | Pre | 5m | 1h | 4h | 24h | Pre | 5m | 1h | 4h | 24h | Pre | 5m | 1h | 4h | 24h | Pre | 5m | 1h | 4h | 24h |  |  |  |  |
| IL-6 | F | 1.71 ± 0.65 | 2.84 ± 1.39^ae^ | 2.11 ± 0.78^ae^ | 2.52 ± 1.56^ae^ | 1.64 ± 0.65 | 1.92 ± 0.87 | 2.56 ± 0.85^ae^ | 3.06 ± 1.72^ae^ | 3.58 ± 2.28^ae^ | 1.77 ± 0.58 | 1.48 ± 0.53 | 2.09 ± 0.55^ae^ | 2.13 ± 1.05^ae^ | 2.58 ± 1.31^ae^ | 1.42 ± 0.32 | 1.5 ± 0.6 | 2.29 ± 0.94^ae^ | 2.61 ± 1.61^ae^ | 2.7 ± 1.22^ae^ | 1.48 ± 0.53 | **<0.001** | **0.003** | 0.353 | **0.003** |
|  | M | 2.03 ± 1.1 | 3.5 ± 2.66^ae^ | 2.54 ± 1.34^ae^ | 2.11 ± 0.83^bce^ | 2.02 ± 0.77 | 3.07 ± 2.8 | 3.58 ± 1.97^ae^ | 3.69 ± 1.66^ae^ | 2.94 ± 2.05^bce^ | 1.72 ± 0.66 | 1.65 ± 0.35 | 2.76 ± 0.76^ae^ | 2.72 ± 0.8^ae^ | 2.04 ± 0.64^bce^ | 1.5 ± 0.45 | 2.09 ± 0.89 | 3.35 ± 1.49^ae^ | 4.96 ± 3.41^ae^ | 2.28 ± 1.47^bce^ | 1.7 ± 0.67 |  |  |  |  |
| IL-8 |  | 5.33 ± 1.1 | 5.97 ± 1.6^a^ | 6.04 ± 1.58^abde^ | 5.16 ± 1.12 | 5.81 ± 2.71 | 5.26 ± 1.2 | 5.72 ± 1.5^a^ | 6.25 ± 1.59 ^abde^ | 5.92 ± 1.24 | 5.38 ± 1.16 | 5.35 ± 1.32 | 5.98 ± 1.65^a^ | 6.16 ± 1.66 ^abde^ | 5.28 ± 1.76 | 5.34 ± 1.48 | 5.38 ± 1.34 | 5.67 ± 1.1^a^ | 6.35 ± 1.29 ^abde^ | 5.63 ± 1.37 | 5.81 ± 1.39 | **<0.001** | 0.279 | 0.551 | 0.871 |
| IFN-γ |  | 0.87 ± 0.35 | 0.73 ± 0.27^a^ | 0.65 ± 0.27^ae^ | 0.64 ± 0.23^ae^ | 0.8 ± 0.27 | 0.92 ± 0.33 | 0.77 ± 0.29^a^ | 0.71 ± 0.28^ae^ | 0.67 ± 0.27^ae^ | 0.99 ± 1.01 | 0.82 ± 0.41 | 0.81 ± 0.88^a^ | 0.82 ± 1.01^ae^ | 0.77 ± 0.72^ae^ | 0.83 ± 0.52 | 0.86 ± 0.39 | 0.7 ± 0.3^a^ | 0.67 ± 0.26^ae^ | 0.69 ± 0.26^ae^ | 0.71 ± 0.22 | **<0.001** | 0.118 | 0.920 | 0.671 |
| TNF |  | 7.48 ± 1.69 | 6.68 ± 1.64^†^ | 6.97 ± 1.55 | 6.8 ± 1.54 | 7.31 ± 1.38^*^ | 7.75 ± 1.9^e^ | 7.8 ± 1.92^§e^ | 7.71 ± 1.77^§e^ | 7.53 ± 1.96^e^ | 7.47 ± 2.03^*‡§^ | 7.09 ± 1.66 | 7.4 ± 1.63^¶^ | 7.56 ± 1.71^¶^ | 7.28 ± 1.69^¶^ | 7.04 ± 1.44^‡^ | 7.52 ± 1.56 | 7.39 ± 1.9^†§¶a^ | 7.72 ± 1.62^§¶^ | 7.27 ± 1.52^§¶a^ | 8.6 ± 2.29^§^ | **0.018** | **<0.001** | **<0.001** | 0.890 |
| IL-1ra |  | 170 ± 35.9 | 189 ± 57.2^e^ | 190 ± 51.2^abe^ | 178 ± 37.5^abe^ | 165 ± 43.1 | 178 ± 45.9 | 177 ± 49.1^e^ | 191 ± 52.9^abe^ | 187 ± 44.2^abe^ | 171 ± 48.3 | 152 ± 26.7 | 174 ± 39.6^e^ | 180 ± 46.1^abe^ | 207 ± 82.6^abe^ | 154 ± 36.7 | 180 ± 45.9 | 185 ± 51.7^e^ | 212 ± 62.5^abe^ | 195 ± 43.1^abe^ | 181 ± 59.5 | **<0.001** | **0.001** | 0.338 | 0.697 |
| IL-10 |  | 2.02 ± 0.37 | 1.72 ± 0.44^*#†^ | 2.52 ± 1.1 | 1.86 ± 0.33^*#b^ | 2.17 ± 0.63^*†abc^ | 2.38 ± 0.47 | 2.53 ± 0.7^*^ | 2.35 ± 0.65^ab^ | 2.11 ± 0.41^*§b^ | 2.03 ± 0.48^*ab^ | 2.1 ± 0.47 | 2.28 ± 0.75^#^ | 2.61 ± 0.87^abe^ | 2.38 ± 0.44^#¶b^ | 2.12 ± 0.41 | 2.03 ± 0.43 | 1.91 ± 0.43^†a^ | 2.34 ± 0.7^abd^ | 2.31 ± 0.62^§¶^ | 2.39 ± 0.59^†bd^ | **<0.001** | **<0.001** | **<0.001** | 0.229 |
| TNF/IL-10 |  | 3.78 ± 0.93 | 4.02 ± 1.06^*#†^ | 3.01 ± 0.91^#†^ | 3.7 ± 0.86^*#^ | 3.54 ± 0.85^b^ | 3.35 ± 0.9 | 3.21 ± 0.82^*^ | 3.39 ± 0.78^§b^ | 3.61 ± 0.92^*abe^ | 3.74 ± 0.83 | 3.45 ± 0.71 | 3.43 ± 0.98^#^ | 3.07 ± 0.89^#ab^ | 3.09 ± 0.61^#b^ | 3.41 ± 0.85 | 3.77 ± 0.73^c^ | 3.94 ± 0.95^†ce^ | 3.46 ± 0.85^†§^ | 3.3 ± 0.89^c^ | 3.67 ± 0.78^c^ | **<0.001** | **0.003** | **<0.001** | 0.215 |

Note: Linear mixed models examining fixed effects of Time*Trial*Sex, a random effect of Participant ID, and trial order as a covariate were conducted. *p*-values are reported for the main effects and interactions, significant *p*-values are bolded. To ensure that trial comparisons (main effect of trial, Trial|Time contrasts, and Trial|Sex contrasts) were not confounded by differences at pre-exercise, a second adjusted model controlling for pre-exercise values that included only post-exercise timepoints (5m, 1h, 4h and 24h) was conducted. Between-trial differences at specific timepoints are denoted with symbols: * = CHO vs. MILK, # = CHO vs. GY, † = CHO vs. WATER, § = MILK vs. WATER, ‡ = MILK vs. GY, ¶ = GY vs. WATER. Within-trial differences between timepoints are denoted with letters: a = difference from Pre, b = difference from 5m, c = difference from 1h, d = difference from 4h, e = difference from 24h. Statistical significance was p≤0.05. Values are mean ± SD. Abbreviations: IL: interleukin; IFN-γ: interferon-γ; TNF: tumor necrosis factor; IL-1ra: interleukin 1 receptor antagonist; M: males; F: females; CHO: carbohydrate; GY: Greek yogurt.
